# The Effectiveness of Behavior Change Techniques Underpinning Psychological Interventions to Improve Glycemic Levels for Adults With Type 2 Diabetes: A Meta-Analysis

**DOI:** 10.3389/fcdhc.2021.699038

**Published:** 2021-07-12

**Authors:** Rebecca Upsher, Deborah Onabajo, Daniel Stahl, Khalida Ismail, Kirsty Winkley

**Affiliations:** ^1^ Department of Psychological Medicine, Institute of Psychiatry, Psychology and Neuroscience, King’s College London, London, United Kingdom; ^2^ Department of Biostatistics, Institute of Psychiatry, King’s College London, London, United Kingdom; ^3^ Florence Nightingale Faculty of Nursing and Midwifery, King’s College London, James Clerk Maxwell Building, London, United Kingdom

**Keywords:** type 2 diabetes, psychological treatment, behavior change, systematic review & meta-analysis, randomized control trial (RCT)

## Abstract

An existing systematic review and meta-analysis found a significant reduction in glycemic levels for adults with type 2 diabetes who received a psychological intervention over control conditions. To help develop effective interventions in the future, there is a need to understand the active ingredients which underpin these psychological interventions. We conducted a secondary meta-analysis including 67 randomized controlled trials (RCTs) reported in English. We reviewed the psychological intervention descriptions of the included studies of the existing review and extracted the behavior change techniques (BCTs) according to the BCT taxonomy (BCTTv1). We also extracted information on primary behavioral target versus primary outcome, and presence of fidelity assessment. The most frequent BCTs across RCTs were ‘social support (unspecified)’ (n=50), ‘problem solving’ (n=38) and ‘goal setting (behavior’) (n=30). These BCTs were independently associated with a significant reduction in glycemic levels (HbA1c) compared to control conditions, but not significantly different from studies that did not include these BCTs. Meta-regressions revealed no significant associations between HbA1c, and psychological intervention category (counselling versus cognitive behavioral therapy interventions) (p=0.84), frequency of BCTs per psychological intervention (p=0.29), primary behavioral target versus primary outcome (p=0.48), or presence of fidelity assessment (p=0.15). Social support (unspecified), problem solving, and goal setting (behavior) could be useful BCTs to develop psychological interventions for people with type 2 diabetes to improve glycemic levels. However, more research is required to understand which combination of individual BCTs are most effective for this population.

**Systematic Review Registration:**

Registered with the international prospective register of systematic reviews registration (PROSPERO) CRD42016033619.

## Introduction

Psychological factors such as depressive symptoms (1), anxiety (2), and diabetes distress (3) can negatively impact type 2 diabetes self-management activities such as diet, exercise, optimal medication-taking behavior, and self-monitoring blood glucose. Psychological interventions are offered to people with type 2 diabetes to address these psychological factors to improve self-management. Optimal self-management of type 2 diabetes leads to normalizing glycaemia to reduce the risk of life-changing long-term diabetes complications (4). Systematic reviews of randomized controlled trials (RCTs) provide evidence that psychological interventions such as cognitive behavioral therapy and counselling (e.g. motivational interviewing) are associated with an improvement in glycemic levels for people with type 2 diabetes (5–8). In these reviews’ facilitators were trained in psychological techniques (an inclusion criteria of studies e.g. cognitive behavioral therapy, motivational interviewing etc.). Psychological interventions differ from educational or behavioral interventions for people with type 2 diabetes, for example DESMOND (9) and X-PERT (10), where facilitators are not trained in psychological techniques and therefore might not be able to adequately address psychological problems that are barriers to optimal self-management. Behavioral interventions predominately target behavior, and not always psychological issues. However, due to the nature of diabetes self-management, psychological interventions not only aim to address psychological issues, but often target behavior as well. Therefore, it is likely that psychological techniques and behavior change techniques are present in psychological interventions.

The most recent of these reviews, Winkley et al. (5), was a systematic review and meta-analysis of psychological interventions to improve glycemic levels (HbA1c) in adults with type 2 diabetes, searching the literature from 2003-2018. N= 94 RCTs met eligibility criteria and n=70 studies were suitable for pooling. There was a small significant reduction in HbA1c (SMD=−0.19, 95% CI =−0.25 to −0.12). This had limited clinical effectiveness in improving HbA1c, an absolute reduction of 3.7 mmol/mol, where 4 mmol/mol is the consensus minimal reduction to reduce risk of long-term diabetes complications (11). Authors speculate there is an issue with limited fidelity assessment, so we do not know whether psychological techniques are delivered as intended, or whether intervention facilitators were competent at delivering these techniques. Additionally, there is need to examine the primary target focus of each individual study as well as understand the specific components of the interventions i.e. the active ingredients present in the psychological intervention.

Psychological interventions such as counselling (e.g. motivational interviewing) or cognitive behavioral therapy, as coded in Winkley et al. (5), are potentially broad in definition. Coding psychological interventions using more specific components such as behavior change techniques (BCTs) increases the probability that future treatments will be more effective owing to certainty around which techniques are the active ingredients of the intervention (12). BCTs can be defined as small, observable and replicable components of an intervention which can lead to a change in behavior in an individual or group of people. The Behavior Change Technique Taxonomy version 1 (BCTTv1) comprises of 93 BCTs (12). The BCTTv1 is a hierarchical taxonomy developed with 54 experts from 7 countries (from psychology, behavioral medicine and health promotion fields) through a series of consensus exercises. The use of these BCTs allows for a standardized language amongst health researchers and healthcare professionals when designing interventions and reporting findings. This ensures that the interventions can be replicated, therefore improving fidelity of delivery, and evaluation (13). There is limited understanding of which BCTs underpin psychological interventions aiming to improve glycemic levels for people with type 2 diabetes. Identifying these BCTs in the intervention design would mean intervention facilitators are more likely to be trained in these techniques, therefore techniques are more likely to be delivered as intended (14). This would then allow intervention developers to evaluate whether these active ingredients incorporated in their intervention are effective in reducing glycemic levels in people with type 2 diabetes.

There is some literature around BCTs and interventions to improve outcomes in people with type 2 diabetes. Studies found that higher frequency of BCTs used in behavioral interventions targeting physical activity and weight loss in type 2 diabetes was associated with greater improvement in glycemic levels (15) and weight loss (16). BCTs in dietary focused interventions that are associated with improved glycemic levels include: ‘instruction on how to perform a behavior’, ‘behavioral practice/rehearsal’, ‘demonstration of the behavior’, and ‘action planning’ (17). BCTs associated with reduced fat intake in type 2 diabetes were associated with ‘goals and planning’, including ‘goal setting’ and ‘review of behavior/outcome goals’ (16). A web-based intervention for people with type 2 diabetes which used the following BCTs were associated with improvements in behavior change, well-being or clinical parameters: ‘feedback on behavior’, ‘information about health consequences’, ‘problem solving’, and ‘self-monitoring of behavior’ (18). A qualitative analysis extracting BCTs from implementation interventions for people with type 2 diabetes (19) based on studies identified in a systematic review (20) found the most frequent BCT categories included: associations, natural consequences, shaping knowledge, antecedents, social support and goals and planning.

The current study was a secondary analysis of the Winkley et al. (5) systematic review and meta-analysis of psychological intervention which aimed to improve glycaemic levels for people living with type 2 diabetes. The Winkley et al. (5) review did not extract information on BCTs underpinning the psychological interventions, primary target behavioral domain, or fidelity assessment.

We set out the following objectives to expand on Winkley et al. (5)’s findings with the aim of examining which components of a psychological intervention are associated with improved HbA1c:

- To code individual BCTs described in each individual study’s psychological intervention description, and to examine whether individual BCTs are associated with an improvement in HbA1c.
- To extract the primary target behavioral domain of each study against its primary outcome, and whether these groupings are associated with improvement in HbA1c.
- To extract how many studies reported fidelity assessment and examine whether presence of fidelity assessment was associated with improved HbA1c.

## Methods

This study extracts individual BCTs from psychological intervention descriptions of an existing systematic review and meta-analysis (5) which was reported according to PRISMA guidelines and registered with PROSPERO, CRD42016033619. This study reports additional analyses which have not been previously reported.

The independent variables were individual BCTs (described at (https://digitalwellbeing.org/wp-content/uploads/2016/11/BCTTv1_PDF_version.pdf), frequency of BCTs per RCT, primary target behavioral domain versus primary outcome, presence of fidelity assessment, and the dependent variable was glycemic levels (HbA1c). Included studies were worldwide RCTs (n=67) reported in English from a published aggregate meta-analysis conducted between 2003-2018 (5).

### The Initial Review

A detailed method section of the original review is reported elsewhere (5). Here we summarize describe characteristics of the original review:

#### Inclusion and Exclusion Criteria

In brief, the review included RCTs comparing psychological interventions (cognitive behavioral therapy, counselling e.g. motivational interviewing, and interpersonal psychotherapy) with a control intervention (usual care, attention control, waiting list, and diabetes education) measuring change in glycemic levels, HbA1c mmol/mol or %, in adults with type 2 diabetes. Data was extracted according to TIDieR guidelines (21) i.e. brief name, WHAT, WHY, WHO, HOW, WHERE, WHEN, HOW MUCH, and HOW WELL of the intervention.

#### Search Strategies

In brief, 6 databases were searched (MEDLINE, EMBASE, PsychINFO, Web of Science, CINAHL, CENTRAL) from January 2003 to July 2018, in addition to conference abstracts (Diabetes UK, American Diabetes Association, European Association for the Study of Diabetes, and International Diabetes Federation), ClinicalTrials.gov, and reference lists of included studies. Key terms were based on ‘diabetes mellitus’, ‘psychological therapies’ and ‘clinical trials’. Some of the included studies had behavioral or educational components, however, all included studies were defined as psychological based on the following criteria: 1) there was a therapeutic alliance between the intervention facilitator and person with type 2 diabetes; 2) the intervention facilitator had been trained in psychological techniques; and 3) the intervention was underpinned by psychological theory. Screening (title/abstract and full text) and data extraction was conducted by two researchers (RU & KW) and discrepancies resolved by a third (KI). References were managed in Endnote X8.

### Secondary Analysis

#### Data Extraction

For this secondary analysis, two researchers (RU & DO) performed data extraction. We extracted individual BCTs from psychological intervention descriptions of studies (reported in English) included in the aggregate meta-analysis of the original review (5) and recorded the number of individual BCTs per study. To further assess mechanisms of action and heterogeneity of psychological interventions reported in the original review (5), for this paper we extracted the target behavioral domain (i.e. behavioral domain being targetted in the intervention, for example, diabetes self-management behaviors) against primary outcome of each individual study, which is recommended in the BCTTv1 training (22). For each individual study, we extracted whether a fidelity assessment was reported. Details on quality assessment are reported elsewhere (5).

#### BCT Coding Procedures

A data extraction table was prepared in Microsoft Excel. The researchers (RU & DO) were health psychology post-graduates and were trained *via* the online BCTTv1 training (22). BCT extraction was pilot tested on 10 studies independently and initial ratings were compared by researchers (RU & DO) to agree on interpretations and prevent future discrepancies. These studies were re-rated, and the remaining studies were independently coded before overall ratings were compared. A third researcher (KW) resolved any disagreements regarding individual BCT coding. Inter-rater reliability between the two researchers’ coders was calculated to determine agreeability and this was high (Cohen’s kappa=0.96).

The psychological intervention description was examined in detail from sources available: published papers, supplementary materials, or study protocols. From intervention descriptions, relevant individual BCT descriptions were copied into the data extraction table. The BCTTv1 was reviewed several times to identify correct individual BCTs to match the language used in the intervention description. In some cases, it was relevant to code more than one individual BCT to an intervention excerpt. Also, multiple examples from an intervention excerpt could be applied to one individual BCT. The BCT must have been related to the intervention target behavior or outcome hence BCTs were not coded with reference to research activity e.g. material reward for taking part in research (as opposed to material reward for engaging in the specific target behavior such as physical activity). Individual BCTs could be extracted from tables outlining interventions, in these cases full phrases or sentences were not extracted e.g. ‘action-planning’ but table text was required to match individual BCT taxonomy codes to be included (no inferences were made).

We did not extract BCTs from control group conditions as there was not enough detail to do so.

#### Data Analysis

Statistical analysis was conducted in STATA 15 (StataCorp, College Station, TX, USA). To gain enough statistical power for meta-regression analyses, five or more studies were required (23).

Meta-regression (24) was performed to determine the association between treatment effect (HbA1c) and study characteristics (individual BCTs [studies with and without individual BCTs], type of psychological intervention, frequency of BCT per psychological intervention, target behavioral domain versus primary outcome category, fidelity assessment). A sub-group meta-analysis was conducted to determine which individual BCTs were associated with improvement in HbA1c. Effect sizes (Cohen’s d) were pooled in a random effects meta-analysis of the standardized mean difference in HbA1c between baseline and follow-up (with reported 95% confidence intervals [CIs]) between psychological intervention and control group. Random effects models were used as there was an assumption that true effect sizes would vary between studies. Studies which reported more than one effect size, i.e. reported HbA1c at different time points, the time point closest to 12-month follow-up (from baseline) was extracted. Statistical heterogeneity, publication bias and statistical outliers were explored in the previous aggregate meta-analysis publication (5).

Moderator and mediators were not assessed in this secondary analysis. Risk of bias assessment was reported in the original review (5) and we did not control for this in the analyses of this study.

## Results

### Study Selection

**Figure 1:**
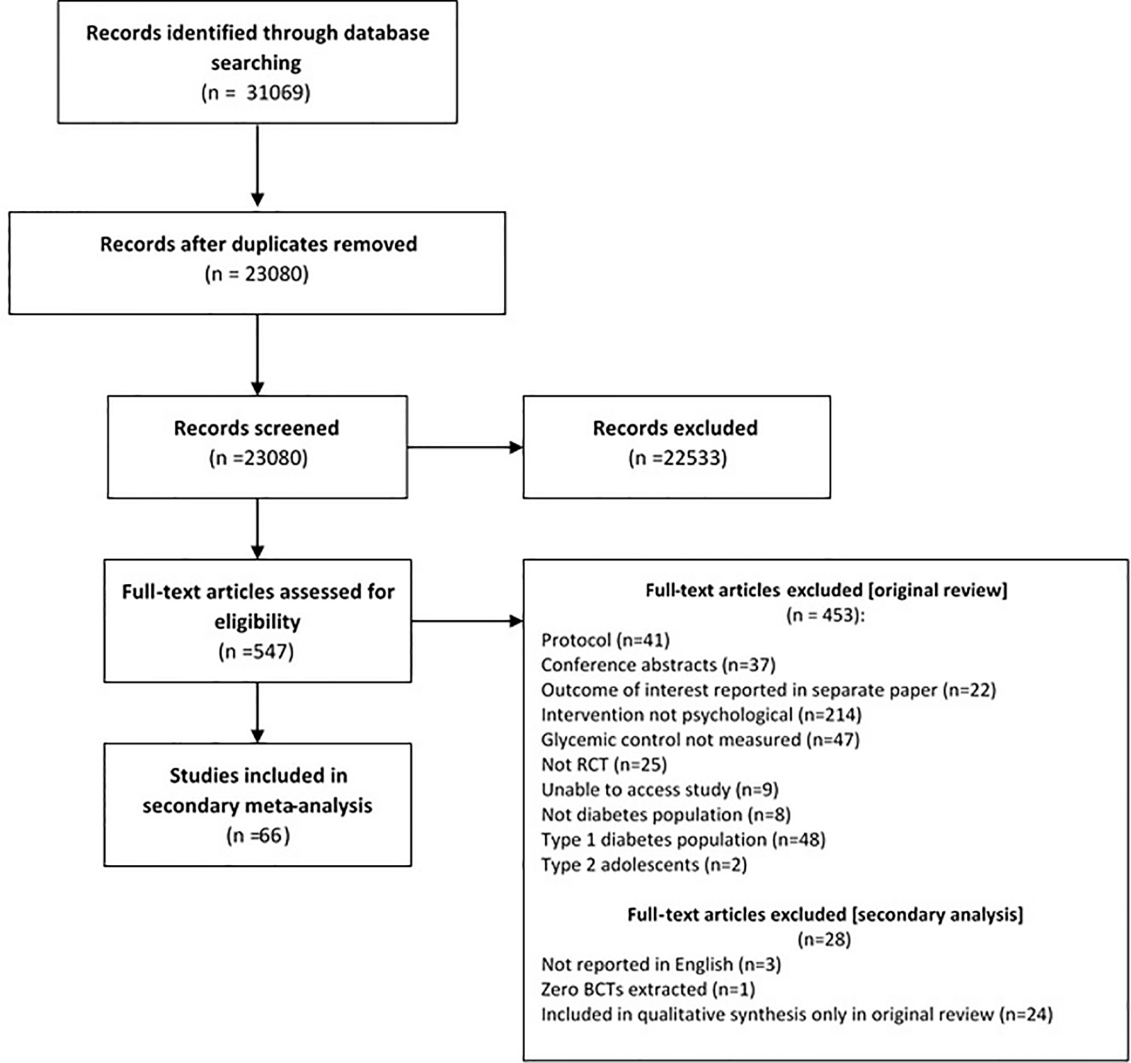
PRISMA flowchart for secondary meta-analysis of psychological interventions to improve glycaemic levels for adults with type 2 diabetes.

Study selection is displayed in **Figure 1**. We analyzed 66 (listed in **Table 1**) out of the 70 studies synthesized in the original review (5). Out of the 70 studies, three studies were excluded as they were not reported in English (translators trained in BCTTv1 unavailable), these studies were reported in Spanish (n=1) (91) and Iranian (n=2) (92, 93). A further study was removed as it did not have sufficient text to describe the intervention and therefore no BCTs were extracted (94). This study was the only study to be categorized as ‘interpersonal therapy’ therefore the remaining studies included in this analysis are categorized as cognitive behavioral therapy or counselling.

**Table 1:** Study characteristics of studies included in meta-analysis of psychological interventions to improve glycaemic levels in type 2 diabetes.

Reference	Brief intervention description	WHAT (Target behavioural domain vs primary outcome)	WHY (type of psychological intervention)	Control group	WHO (provider of intervention)	HOW (Mode, format)	WHERE (country of recruitment)	WHEN and HOW MUCH (number of sessions, duration of intervention)	HOW WELL (fidelity of intervention reported)?
(25)	Empowerment (BATHE technique to increase diabetes self-efficacy)	Mood management vs psychological outcome	Counselling	Usual care	Physicians	Face to face, one-to-one	Europe	3 sessions over 3 months	O
(26)	Behavioral intervention to increase physical activity and reduce sedentary time	Self-management target vs Self-management outcome	Counselling	Usual care	Diabetologists, exercise specialists	Face to face, one-to-one	Europe	9 sessions over 3 years	O
(27)	Motivational interviewing intervention to promote diabetes behavior change to reach treatment goal HbA1c <7%	Self-management target vs HbA1c outcome	Counselling	Usual care	Clinicians (doctors, nurses, psychologists)	Telephone and face to face, one-to-one	Asia	9 sessions over 12 months	O
(28)	Motivational interviewing intervention to promote diabetes behavior change and provide diabetes health education	Self-management target vs HbA1c outcome	Counselling	Enhanced usual care	Community health workers	Telephone and face to face, one-to-one	North America	Variable number of sessions over 12 months	P
(29)	Lifestyle nutrition intervention to increase physical activity	Self-management target vs HbA1c outcome	Counselling	Usual care	Dietician and physician	Face to face, one-to-one	Asia	1 session over 2 weeks	O
(30)	Motivational interviewing intervention to improve diabetes self-management behaviors	Self-management target vs HbA1c outcome	Counselling	Diabetes education	Diabetes nurses	Face to face, one-to-one	Asia	Not reported	O
(31)	Value-based emotion-focused educational programme	Mood management vs psychological outcome	Counselling	Attention control	Nurse and physician	Face to face, group	Asia	4 sessions over 6 weeks	O
(32)	Minimal psychological intervention (MPI) on improving psychological well-being	Mood management vs psychological outcome	Counselling	Usual care	Psychology assistants	Telephone, one-to-one	Asia	4 sessions	O
(33)	Motivational interviewing intervention on diabetes regimen adherence.	Self-management target vs Self-management outcome	Counselling	Usual care	Diabetes nurse	Face to face, one-to-one	North America	4-6 sessions over 3 months	P
(34)	Collaborative care model to treat community mental health centre (CMHC) people with psychosis and suboptimal glycaemic levels.	psychological target vs HbA1c outcome	Counselling	Usual care	Nurse case manager, psychiatrist, advanced practice nurse	Face to face, one-to-one	North America	12 sessions over 9 months	O
(35)	Peer telephone intervention to enhance self-efficacy	Mood management vs psychological outcome	Counselling	Usual care	Diabetes nurses	Telephone, one-to-one	Europe	6 sessions over 150 days	O
(36)	A cognitive-behavioral pedometer-based group intervention on physical activity and sedentary behavior	Self-management target vs Self-management outcome	CBT	Usual care	MSc level coaches (PE or clinical psychology)	Face to face, group	Europe	5 sessions over 12 weeks	O
(37)	Pedometer-based physical activity program	Self-management target vs Self-management outcome	Counselling	Usual care	Clinical psychologist	Face to face, group	Europe	3 sessions over 12 weeks	O
(38)	Telephone-Delivered Lifestyle Support with Action Planning and Motivational Interviewing Techniques to Improve Rehabilitation Outcomes	Self-management target vs Self-management outcome	Counselling	Usual care	Counsellors	Face to face, one-to-one	Europe	12 sessions over 12 months	P
(39)	Telephone Delivered Weight Loss and Physical Activity Intervention	Self-management target vs Biomedical outcome	Counselling	Usual care	Counsellors	Telephone, one-to-one	Australia	27 sessions over 18 months	P
(40)	Psychotherapy for depression *via* home telehealth	Mood management vs psychological outcome	CBT	Same-room treatment	Therapists with 5 years’ experience	Face to face, one-to-one	North America	8 sessions over 8 weeks	P
(41)	Collaborative care intervention to reduce depressive symptoms	Mood management vs psychological outcome	CBT	Enhanced usual care	Primary care physicians, graduate social workers, diabetes depression clinical specialists	Telephone and face to face, one-to-one	North America	Variable number of sessions over 12 months	O
(42)	Group based cognitive behavioral therapy program to improve depression, anxiety and stress	Mood management vs psychological outcome	CBT	Waiting list control	Not reported	Face to face, group	Australia	7 sessions over 3 months	O
(43)	Individualized diabetes education with tailored self-care plan (covering dietary modification, exercises programs, adherence to medications, self-monitoring of blood glucose and blood pressure, and psychological counselling)	Self-management target vs Biomedical outcome	Counselling	Group education	Nurses, clinical psychologists	Face to face, group	Asia	3 sessions over 3 months	O
(44)	Nurse-led intervention to support people with type 2 diabetes with adherence to taking glucose lowering medication	Self-management target vs Self-management outcome	Counselling	Usual care	Clinical nurses	Face to face, one-to-one	Europe	1 session over 1 day	P
(45)	Novel model of care (“Stepping Up”) intervention in normalising insulin initiation for type 2 diabetes	Self-management target vs HbA1c outcome	Counselling	Usual care	Registered nurses	Face to face, one-to-one	Australia	Variable number of sessions over 12 months	O
(46)	Culturally sensitive family-oriented intervention to discuss family or other psychosocial factors that could interfere with their diabetes control.	psychological target vs HbA1c outcome	Counselling	Usual care	Healthcare team	Face to face, family	South America	4 sessions over 12 months	O
(47)	Family social support to stimulate dialogue between person with diabetes and family to increase interest and assistance in achieving diabetes self-management goals	Self-management target vs HbA1c outcome	Counselling	Education	Family	Telephone, family	South America	4 sessions over 9 months	O
(48)	Acceptance and commitment therapy to improve diabetes self-management	Self-management target vs HbA1c outcome	CBT	Education alone	Psychologist	Face to face, group	North America	1 session over 1 day	O
(49)	Theory-based behavior change intervention to improve physical activity, dietary change, medication adherence and smoking cessation	Self-management target vs Self-management outcome	Counselling	Intensive treatment alone	Lifestyle facilitator	Telephone and face to face, one-to-one	Europe	8 sessions over 1 year	P
(50)	Mindfulness-Based Stress-Reduction Intervention	Mood management vs psychological outcome	Counselling	Usual care	Psychologist, resident in internal medicine	Face to face, group	Europe	8 sessions over 8 weeks	O
(51)	Videophone Motivational Diabetes Self-Management Intervention	Self-management target vs HbA1c outcome	Counselling	Attentional control	Nurse practitioner	Telephone, one-to-one	North America	12 sessions over 3 months	P
(52)	Diabetes-Specific Cognitive Behavioral Treatment Program (DIAMOS) for Patients with Diabetes and Subclinical Depression	Mood management vs psychological outcome	CBT	Diabetes education	Psychologist	Face to face, group	Europe	5 sessions over variable time period	O
(53)	A self-management-oriented education programme (MEDIAS 2 BSC) for people with Type 2 diabetes who are on a non-intensive insulin treatment regimen	Self-management target vs HbA1c outcome	Counselling	Diabetes education	Diabetes educators	Face to face, group	Europe	6 sessions over 6 weeks	O
(54)	Motivational enhancement therapy plus cognitive behavior therapy on depressive symptoms and health-related quality of life in adults with type 2 diabetes	psychological target vs HbA1c outcome	CBT	Usual care	Psychotherapist, clinical nurse	Face to face, group	Asia	12 sessions over 3 months	O
(55)	Nurse-led motivational interviewing plus cognitive behavioral therapy intervention to change and address barriers to diabetes self-management behaviors	Self-management target vs HbA1c outcome	Counselling	Usual care	Nurses	Face to face, one-to-one	Europe	12 sessions over 12 months	P
(56)	Lifestyle counselling based on motivational interviewing principles to improve diabetes care	Self-management target vs HbA1c outcome	Counselling	Usual care	Primary care nurse	Face to face, one-to-one	Europe	5-8 sessions over 6 months	O
(57)	Care intervention including dietary intervention, exercise intervention, and psychology intervention	Self-management target vs HbA1c outcome	Counselling	Usual care	Dieticians, psychologists	Telephone and face to face, group	Asia	12 sessions over 12 months	O
(58)	Self-determination intervention for general practice nurses to improve care in people with type 2 diabetes	Self-management target vs HbA1c outcome	Counselling	Usual care	General practitioner nurses	Face to face, one-to-one	Europe	Not reported	O
(59)	Theory-based health promotion intervention to improve health behavior	Self-management target vs Biomedical outcome	Counselling	Usual care	Dietician, occupational therapist	Face to face, group	Europe	6 sessions over 6 months	O
(60)	Tailored, supportive intervention strategy to increasing self-efficacy and improving illness perceptions in people with type 2 diabetes shortly after a first acute coronary event.	Mood management vs psychological outcome	Counselling	Attentional control	Diabetes nurses	Face to face, one-to-one	Europe	3 sessions over 2.5 months	O
(61)	Self-management program for Thais with type 2 diabetes	Self-management target vs HbA1c outcome	CBT	Diabetes education	Diabetes researcher	Face to face, group	Asia	5 sessions over 2 weeks	O
(62)	Psychological Family Intervention to improve diabetes self-management and mobilise family support	Self-management target vs HbA1c outcome	Counselling	Usual care	Health psychologist	Face to face, family	Europe	3 sessions over 3 weeks	O
(63)	Community-based, culturally tailored, multimodal behavioral intervention in an ethic/linguistic minority group with type 2 diabetes	Self-management target vs HbA1c outcome	Counselling	Education only	Nurses, community health workers	Face to face, group	North America	6 sessions over 6 weeks	P
(64)	Nurse-administered minimal psychological intervention for depressive symptoms	mood management vs psychological outcome	CBT	Usual care	Primary care nurse	Face to face, one-to-one	Europe	Variable number of sessions over variable time period	P
(65)	Motivational interviewing intervention focused on behavior change	Self-management target vs HbA1c outcome	Counselling	Diabetes education	Therapist	Face to face, one-to-one	Asia	4 sessions over 6 months	O
(66)	Music therapy to improve diabetes self-management	Self-management target vs HbA1c outcome	CBT	Diabetes education	Music therapist	Face to face, group	North America	4 sessions over 8 weeks	P
(67)	Culturally relevant group diabetes self-management training (DSMT), coping skills training (CST), and diabetes care intervention	Self-management target vs HbA1c outcome	CBT	Usual care	Diabetes nurses	Face to face, group	North America	11 sessions over 11 weeks	P
(68)	Group motivational interviewing therapy to promote positive lifestyle changes	Self-management target vs HbA1c outcome	Counselling	Wait-list control	Psychiatrist	Face to face, group	Asia	4 sessions over 4 weeks	O
(69)	A brief culturally tailored intervention for Puerto Ricans with type 2 diabetes to promote health behavior change	Self-management target vs Self-management outcome	Counselling	Usual care	Medical assistant (trained by diabetes educator)	Face to face, one-to-one	North America	1 session over 1 day	O
(70)	Psychoeducational Intervention (SWEEP) for Depressed Women with Diabetes	Mood management vs psychological outcome	CBT	Usual care	CBT trained nurse	Face to face, group	North America	8 sessions over 8 weeks	P
(71)	Cognitive behavioral therapy people with diabetes and depression	psychological target vs HbA1c outcome	CBT	Sertraline treatment + usual care	Clinical psychologist	Face to face, group	Europe	10 sessions over 12 weeks	O
(72)	Psychoeducation and physical exercise for people with type 2 diabetes and subsyndromal depression.	Mood management vs psychological outcome	CBT	Enhanced usual care	Psychologist	Face to face, group	Europe	6 sessions over 6 weeks	O
(73)	Telephonic counselling plus walking for depressed people with type 2 diabetes	psychological target vs HbA1c outcome	CBT	Enhanced usual care	Nurse	Telephone, one-to-one	North America	12 sessions over 12 months	P
(74)	Motivational interviewing intervention to improve medication adherence	Self-management target vs HbA1c outcome	Counselling	Usual care	Diabetes nurses, pharmacists	Telephone and face to face, one-to-one	North America	6 sessions over 18 months	P
(75)	Theory-based intervention to increase physical activity in adults with type 2 diabetes	Self-management target vs HbA1c outcome	Counselling	Physical activity education materials	Individuals with a degree in physical activity promotion/counselling	Telephone, one-to-one	North America	22 sessions over 18 months	O
(76)	Problem-solving therapy for adults with diabetic retinopathy and diabetes specific distress	Mood management vs psychological outcome	CBT	Usual care	Research assistant trained in problem solving therapy	Telephone and face to face, one-to-one	Australia	8 sessions over variable time period	O
(77)	A brief telephone coaching intervention to promote diabetes self-management	Self-management target vs Self-management outcome	Counselling	Usual care	Undergraduate psychologist	Telephone, one-to-one	North America	18 sessions over 6 months	O
(78)	Cognitive behavioral therapy for medication adherence and depression	Self-management target vs Self-management outcome	CBT	Enhanced usual care	Therapist	Face to face, one-to-one	North America	9-12 sessions over 4 months	O
(79)	Acceptance and Commitment Therapy for type 2 diabetes management	Self-management target vs HbA1c outcome	CBT	Education with routine treatment	Clinical psychologist	Face to face, group	Asia	10 sessions over 3 months	O
(80)	Self-monitoring blood glucose intervention	Self-management target vs HbA1c outcome	Counselling	Non-standardised counselling	Physician	Face to face, one-to-one	Europe	4 sessions over 24 weeks	O
(81)	Self-management intervention for type 2 diabetes	Self-management target vs psychological outcome	Counselling	Usual care	Diabetes specialist nurses, dieticians	Face to face, group	Europe	5 sessions over 5 weeks	O
(82)	Mindfulness-based cognitive therapy for people with diabetes and emotional problems	Mood management vs psychological outcome	CBT	Usual care	Psychologist	Face to face, group	Europe	8 sessions over 8 weeks	O
(83)	Stress management intervention for Latinos with type 2 diabetes	Mood management vs psychological outcome	Counselling	Diabetes education;	Community health worker	Face to face, one-to-one	North America	8 sessions over 10 weeks	P
(84)	Motivational interviewing diabetes self-management education intervention to improve behavior change	Self-management target vs HbA1c outcome	Counselling	Diabetes self-management education	Diabetes educators	Face to face, one-to-one	North America	4 sessions over 6 months	P
(85)	Individualised cognitive behavioral treatment to promote behavior change	Self-management target vs Biomedical outcome	CBT	Usual care	Diabetes nurse, dietician	Face to face, one-to-one	Europe	3-6 sessions over variable time period	P
(86)	Motivational interviewing to improve weight loss	Self-management target vs Biomedical outcome	Counselling	Attention control	Clinical psychologist	Face to face, one-to-one	North America	5 sessions over 12 months	P
(87)	Nurse-coaching intervention for women with type 2 diabetes to integrate diabetes self-management into their daily lives	Self-management target vs Self-management outcome	Counselling	Usual care	Nurses	Face to face, one-to-one	North America	6 sessions over 6 months	O
(88)	Collaborative treatment for depression and diabetes	psychological target vs HbA1c outcome	CBT	Usual care	Depression clinical specialist	Face to face, one-to-one	North America	7 sessions over variable time period	O
(89)	Integrative health coaching for people with type 2 diabetes	Self-management target vs Self-management outcome	Counselling	Usual care	MSc level coaches (in social work or psychology)	Face to face, one-to-one	North America	14 sessions over 6 months	O
(90)	Cognitive behavioral therapy focusing on depression and anxiety	Mood management vs psychological outcome	CBT	Usual care	IAPT practitioners	Face to face, group	Europe	6 sessions over 6 weeks	O

*CBT, cognitive behavioral therapy; IPT, Interpersonal psychotherapy NR, not reported.

### Study Characteristics

All study characteristics are reported in **Table 1**, and more detail on study characteristic group categorizations and characteristics of individual studies are reported in the original review (5). Studies were conducted in Europe (n=25), North America (n=23), Asia (n=12), Australia (n=4), or South America (n=2).

#### Population

All studies reported psychological interventions targeting people living with type 2 diabetes (N=66).

#### Psychological Interventions

There were more counselling studies (n=44) than cognitive behavioral therapy (n=22) psychological interventions. Psychological interventions were delivered by diabetes specialists (n=30), psychology professionals (n=21), and other facilitators (n=14). The target behavioral domain of interventions was categorized as mood management (n=23) or diabetes self-management (n=43).

#### Control Condition

Control groups were usual care (n=48), attention control (n=15), diabetes education (n=3).

#### Outcome

The primary outcomes of individual studies were HbA1c (n=31), self-management (n=12), psychological (n=18), and biomedical (n=5).

### Synthesis of Results

#### BCT Coding

Examples of how each BCTs were coded from psychological intervention descriptions is reported in **Table S1**. The following website provides BCT definitions and examples according to the BCTTv1: (https://digitalwellbeing.org/wp-content/uploads/2016/11/BCTTv1_PDF_version.pdf).

Individual BCTs which were reported in 5 or more studies (19 BCTs) are reported in **Table 2**. Overall, the most common BCT across studies which were associated with a significant reduction in HbA1c compared to the control condition included: ‘social support (unspecified)’ (n=50 RCTs, SMD=-0.17, 95% CI=-0.23, -0.10), followed by ‘problem solving’ (n=38 RCTs, SMD=-0.16, 95% CI=-0.24, -0.08), and ‘goal setting (behavior)’ (n=30 RCTs, SMD=-0.19, 95% CI=-0.31, -0.07), **Table 3**. Other individual BCTs that were associated with a significant reduction in HbA1c compared to the control condition included: ‘goal setting (outcome)’ (n=7, SMD=-0.21, 95% CI=-0.38, -0.03), ‘action planning’ (n=14, SMD=-0.14, 95% CI=-0.27, -0.01), ‘review of behavior goal(s)’ (n=7, SMD=-0.24, 95% CI=-0.40, -0.07), ‘feedback on behavior’ (n=9, SMD=-0.33, 95% CI=-0.64, -0.01), ‘self-monitoring of behavior’ (n=19, SMD=-0.28, 95% CI=-0.45, -0.11), ‘instruction on how to perform the behavior’ (n=18, SMD=-0.24, 95% CI=-0.40, -0.08), ‘reduce negative emotions’ (n=19, SMD=-0.18, 95% CI=-0.30, -0.06), and ‘framing/reframing’ (n=13, SMD=-0.28, 95% CI=-0.53, -0.04), **Table 3**. However, even though psychological interventions which included these individual BCTs were associated with significantly reduced HbA1c over the control condition, there were no significant differences in effect size between studies which included each individual BCT versus studies which did not include each BCT (**Table 3**).

**Table 2:** Number of counselling and cognitive behavioral therapy studies which included each individual BCT.

BCT label	Total number of studies including each individual BCT*	Number of counselling studies (n=44) which included each individual BCT (%)	Number of cognitive behavioral therapy studies (n=22) which included each individual BCT (%)
**1.1 Goal setting (behavior)**	30	20 (45.45)	10 (45.45)
**1.2 Problem solving**	38	23 (52.27)	15 (68.18)
**1.3 Goal setting (outcome)**	7	5 (11.36)	2 (9.09)
**1.4 Action planning**	14	9 (20.45)	5 (22.73)
**1.5 Review of behavior goal(s)**	7	3 (6.82)	4 (18.18)
**2.2 Feedback on behavior**	9	7 (15.91)	2 (9.09)
**2.3 Self-monitoring of behavior**	19	12 (27.27)	7 (31.82)
**2.4 Self-monitoring of outcome(s) of behavior**	6	5 (11.36)	1 (4.55)
**3.1 Social support (unspecified)**	50	35 (79.55)	15 (68.18)
**3.3 Social support (emotional)**	10	8 (18.18)	2 (9.09)
**4.1 Instruction on how to perform the behavior**	18	14 (31.82)	4 (18.18)
**6.1 Demonstration of the behavior**	12	8 (18.18)	4 (18.18)
**8.1 Behavioral practice/rehearsal**	5	4 (9.09)	1 (4.55)
**8.7 Graded tasks**	8	7 (15.91)	1 (4.55)
**9.2 Pros and cons**	8	6 (13.64)	2 (9.09)
**10.3 Non-specific incentive**	5	2 (4.55)	3 (13.64)
**11.2 Reduce negative emotions**	19	5 (11.36)	14 (63.64)
**12.5 Adding objects to the environment**	8	6 (13.64)	2 (9.09)
**13.2 Framing/reframing**	13	7 (15.91)	6 (27.27)

*Where less than 5 studies per BCT were present, this have been removed from this table (as they were not included in meta-analysis).

**Table 3:** Standardised mean difference in glycaemic control per individual BCT.

BCT		N	SMD	95% CI (p-value)	Difference between studies containing BCT vs those without BCT (P-value)
1.1 Goal setting (behavior)					0.80
	With BCT	30	-0.19	-0.31, -0.07 (0.001)	
	Without BCT	36	-0.16	-0.24, -0.09 (<0.001)	
1.2 Problem solving					0.65
	With BCT	38	-0.16	-0.24, -0.08 (<0.001)	
	Without BCT	28	-0.20	-0.30, -0.09 (<0.001)	
1.3 Goal setting (outcome)					0.79
	With BCT	7	-0.21	-0.38, -0.03 (0.025)	
	Without BCT	59	-0.17	-0.24, -0.10 (<0.001)	
1.4 Action planning					0.66
	With BCT	14	-0.14	-0.27, -0.01 (0.03)	
	Without BCT	52	-0.18	-0.26, -0.11 (<0.001)	
1.5 Review of behavior goal(s)					0.46
	With BCT	7	-0.24	-0.40, -0.07 (0.004)	
	Without BCT	59	-0.17	-0.23, -0.10 (<0.001)	
2.2 Feedback on behavior					0.11
	With BCT	9	-0.33	-0.64, -0.01 (0.04)	
	Without BCT	57	-0.15	-0.21, -0.09 (<0.002)	
2.3 Self-monitoring of behavior					0.17
	With BCT	19	-0.28	-0.45, -0.11 (0.001)	
	Without BCT	47	-0.13	-0.20, -0.07 (<0.001)	
2.4 Self-monitoring of outcome(s) of behavior					0.70
	With BCT	6	-0.14	-0.33, 0.06 (0.17)	
	Without BCT	60	-0.18	-0.25, -0.11 (<0.001)	
3.1 Social support (unspecified)					0.79
	With BCT	50	-0.17	-0.23, -0.10 (<0.001)	
	Without BCT	16	-0.21	-0.39, -0.03 (0.02)	
3.3 Social support (emotional)					0.28
	With BCT	10	-0.09	-0.22, 0.04 (0.17)	
	Without BCT	56	-0.19	-0.26, -0.12 (<0.001)	
4.1 Instruction on how to perform the behavior					0.29
	With BCT	18	-0.24	-0.40, -0.08 (0.003)	
	Without BCT	48	-0.14	-0.20, -0.07 (<0.003)	
6.1 Demonstration of the behavior					0.69
	With BCT	12	-0.21	-0.45, 0.04 (0.10)	
	Without BCT	54	-0.16	-0.22, -0.10 (<0.001)	
8.1 Behavioral practice/rehearsal					0.31
	With BCT	5	-0.04	-0.21, 0.14 (0.70)	
	Without BCT	61	-0.19	-0.25, -0.12 (<0.001)	
8.7 Graded tasks					0.84
	With BCT	8	-0.21	-0.49, 0.07 (0.14)	
	Without BCT	58	-0.17	-0.24, -0.11 (<0.001)	
9.2 Pros and cons					0.83
	With BCT	8	-0.15	-0.40, 0.09 (0.22)	
	Without BCT	58	-0.18	-0.24, -0.11 (<0.001)	
10.3 Non-specific incentive					0.06
	With BCT	5	0.08	-0.21, 0.37 (0.57)	
	Without BCT	61	-0.19	-0.26, -0.13 (<0.001)	
11.2 Reduce negative emotions					0.90
	With BCT	19	-0.18	-0.30, -0.06 (0.004)	
	Without BCT	47	-0.17	-0.25, -0.10(<0.001)	
12.5 Adding objects to the environment					0.83
	With BCT	8	-0.21	-0.52, 0.10 (0.18)	
	Without BCT	58	-0.17	-0.23, -0.11 (<0.001)	
13.2 Framing/reframing					0.22
	With BCT	13	-0.28	-0.53, -0.04 (0.02)	
	Without BCT	53	-0.15	-0.21, -0.09 (<0.001)	

The most common individual BCTs in counselling studies were ‘social support’ (unspecified; 79.55%), ‘problem solving’ (52.27%), ‘goal setting’ (behavior; 45.45%), and ‘instruction on how to perform the behavior’ (31.82%), **Table 2**. There were similar most common individual BCTs present in cognitive behavioral therapy studies: ‘social support’ (unspecified; 68.18%), ‘problem solving’ (68.18%), ‘reduce negative emotions’ (63.64%), and ‘goal setting’ (behavior; 9.09%). A meta-regression found no difference in glycemic level effect size between counselling and cognitive behavioral therapy conditions (b=-0.17, 95% CI= -0.18, 0.15, p=0.84).

**Figure 2:**
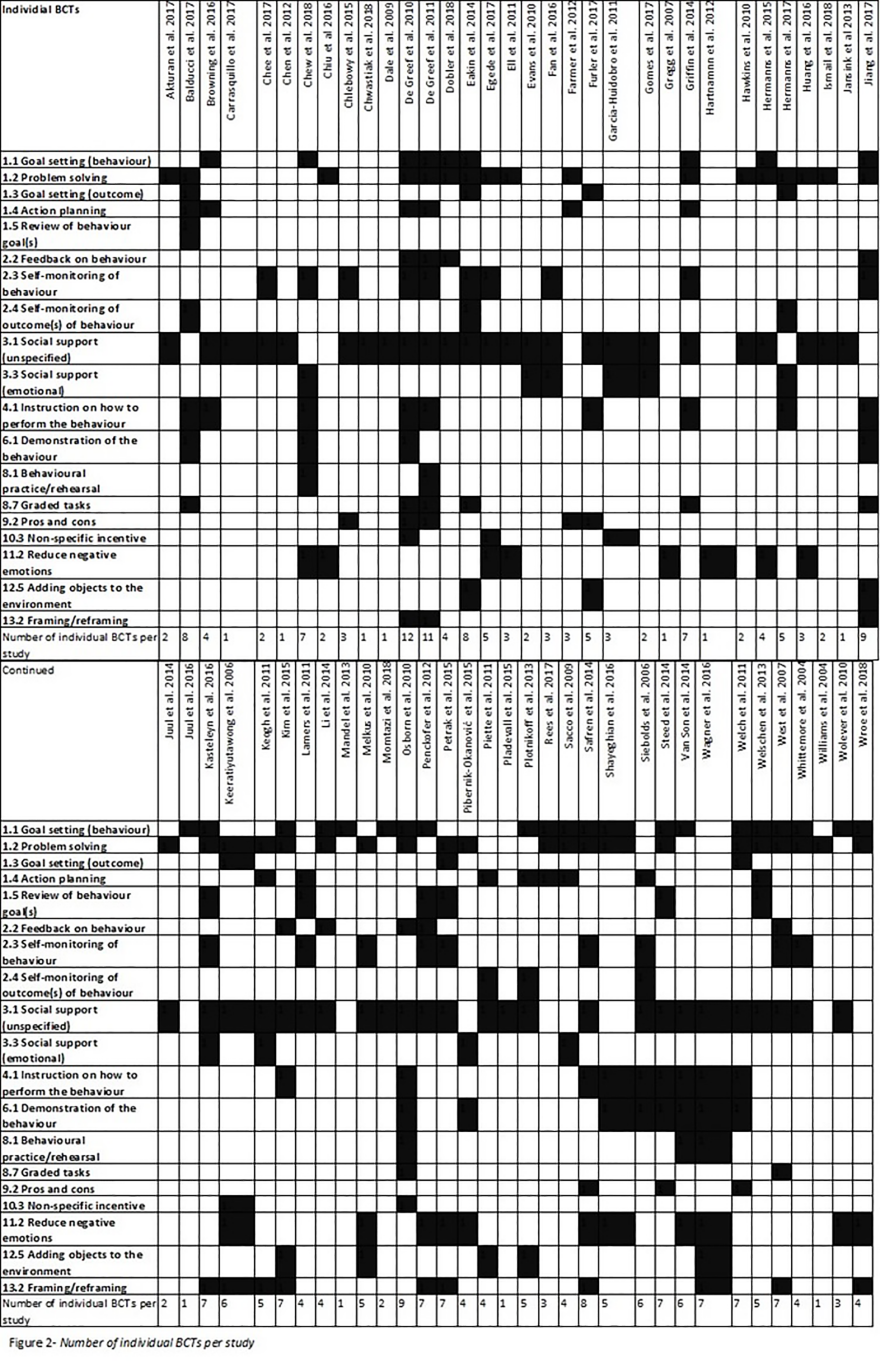
Number of individual BCTs per study.

The range of individual BCTs per psychological interventions was 1-12 (**Figure 2**). One study reported using 12 individual BCTs in their intervention (36). The mean number of individual BCT per psychological intervention was 4.33 (SD=2.65). A meta-regression found no association between HbA1c and, frequency of BCTs per psychological intervention (b=-0.02 [95% CI=-0.05, 0.02], p=0.29).

#### Target Behavioral Domain Versus Primary Outcome Category

The target behavioral domain versus primary outcome were grouped for a meta-regression including the following categories (**Table 1**): mood management versus psychological outcome (n=17), mood management versus HbA1c outcome (n=6), self-management versus self-management outcome (n=12), self-management versus HbA1c (n=25), self-management versus biomedical outcome (n=5), and self-management versus psychological outcome (n=1). A meta-regression found no association between HbA1c and, target behavioral domain versus primary outcome category (b=0.113 [95% CI=-0.29, 0.03], p=0.48).

#### Fidelity Assessment

Fidelity assessment of the psychological interventions was present in 20 out of the 66 studies (**Table 1**) *via* expert observation or assessment of audio-tape recordings of psychological interventions. A meta-regression found no association between HbA1c and, presence of fidelity assessment (b=0.11 [95% CI=-0.04, 0.27], p=0.15).

## Discussion

We conducted a secondary analysis of the Winkley et al. (5) systematic review and meta-analysis of psychological interventions to improve glycemic levels in adults with type 2 diabetes. We further extracted data on BCTs (from psychological intervention descriptions), target behavior domain versus primary outcome, and presence of fidelity assessment.

It was not possible to identify the most effective BCTs (compared to studies which did not include them) to improve glycemic levels for people with type 2 diabetes. However, the most frequently used BCTs which were independently associated with statistically significant improvements in glycemic levels (compared to control conditions) included: ‘social support (unspecified)’, ‘problem solving’, and ‘goal setting’ (behavior)’. These were the 3 BCTs which were most common for both counselling and cognitive behavioral therapy interventions. This may account for why no differences in glycemic level improvement were found between cognitive behavioral therapy and counselling interventions. Another reason for lack of differences between cognitive behavioral therapy and counselling interventions could be based on the categorization of psychological interventions in the Winkley et al. (5) review. Cuijpers et al. (95) discuss how psychological therapies work. For example, *via* specific effects that focus on the therapeutic approach and underlying theoretical model (e.g. cognitive therapy targets maladaptive cognitions, behavioral therapy targets maladaptive behaviors), or *via* common factors which are the commonalities between all therapies (e.g. therapeutic alliance). Cuijpers concluded that there is not enough evidence to determine which approach explains how therapies work. In the Winkley et al. (5) review, studies were included based on the later approach, i.e. common factors, and defined psychological interventions based on them having a therapeutic alliance between intervention facilitator and person living with diabetes. Future work in this area should consider how both therapeutic approach factors and common factors interact in a complex way, perhaps by involving different mediating variables.

Other BCTs which were independently associated with statistically significant improvements in glycemic levels (compared to control conditions) included: ‘goal setting (outcome)’, ‘action planning’, ‘review of behavior goal(s)’, ‘feedback on behavior’, ‘self-monitoring of behavior’, ‘instruction on how to perform the behavior’, ‘reduce negative emotions’, and ‘framing/reframing’. Other research with adults with type 2 diabetes examining the effectiveness of BCTs have similarly found improved outcomes using ‘instruction on how to perform a behavior’, ‘action planning’ (17), ‘goal setting’, ‘review of behavior goals’ (16), ‘feedback on behavior’, ‘problem solving’, ‘self-monitoring of behavior’ (18), and ‘social support’ (19). However, these studies did not investigate the association between psychological interventions and glycemic levels, and therefore our findings make a novel contribution to the literature.

We identified the mean number of individual BCTs per psychological intervention for people with type 2 diabetes was 4.33. There was no association between frequency of individual BCTs per psychological intervention and glycemic levels, therefore the optimal number of individual BCTs in a psychological intervention which improve glycemic levels for people with type 2 diabetes cannot be determined. This is a similar finding to a meta-regression in a study of behavioral interventions for obese adults where more BCTs were not associated with better outcomes (i.e. improving diet and/or physical activity) (96). Whereas other type 2 diabetes research has reported the opposite that the more BCTs used, the better the outcomes, but these did not include glycemic control (15, 16). In our analysis, the trial which reported the highest number of individual BCTs per psychological intervention did not have the largest effect size in improving glycemic levels (36). Again, supporting our conclusion that there is no association between frequency of individual BCTs and glycemic levels.

The Winkley et al. (5) review and this subsequent secondary analysis focused on glycemic levels as an outcome. We felt it did not make sense to exclude studies where glycemic levels are a secondary outcome for this analysis. Therefore, studies with different primary behavioral domain targets (e.g. self-management or mood management) and primary outcomes (HbA1c, self-management or psychological) were included. In our analysis, we grouped studies according to primary target versus primary outcome, and did not find any significant differences in effect size between groups in improving HbA1c. A reason for this could be that regardless of the primary target behavioral domain, e.g. self-management behaviors and mood management, both aim to indirectly improve glycemic levels. For example, a primary target might be physical activity and primary outcome is weight loss, where weight loss leads to decrease in insulin resistance which improves glycemic levels. Another example could be the primary target being mood management and primary outcome is reduction in depressive symptoms, this increases cognitive capacity to engage in self-management behaviors such as optimal medication taking behavior, which then leads to an improvement in glycemic levels.

Therapeutic alliance present in psychological interventions can conceptually separate behavior change interventions from psychological therapies. However, less than a third of studies reported fidelity assessment and therefore we were unable to determine whether intervention facilitators were competent at delivering therapeutic skills and BCTs or whether therapeutic skills and BCTs were delivered as intended. Even though our meta-regression revealed that there was no significant difference in HbA1c improvement between studies which did and did not report fidelity, this is still an issue. Other systematic reviews also note poor reporting of fidelity assessment (97, 98). For one nurse-led diabetes study which did assess fidelity (55), it was found that some psychological techniques were delivered in the control condition (99), indicating contamination of skills can be an issue with RCT results and subsequent interpretation. This also highlights the importance of fidelity assessment, so it is known which skills were delivered in the study conditions.

### Why BCTs Might Be Effective in Psychological Interventions for People With Type 2 Diabetes

It’s important to understand why BCTs might be effective in reducing HbA1c in type 2 diabetes to provide insight for future intervention developers. Here, we focus on the three most common BCTs extracted in our analysis: ‘social support (unspecified)’, ‘problem solving’, and ‘goal setting’ (behavior).’

The positive benefits of social support for people with type 2 diabetes are well documented including improved HbA1c (100, 101), increased diabetes self-management, and optimal medication-taking behavior (100). There are two main hypotheses for why social support has a positive impact on physical and mental health. The ‘buffering hypotheses’ states that social support is protective during stressful events, so if a person has less or no social support then they are more susceptible to the negative impact of a stressful event (102) (e.g. in type 2 diabetes engaging in multiple self-management behaviors). However, ‘direct effects’ hypothesis says that people with high levels of social support have better health, irrespective of a stressful event (102). Both the size of a social support network and the quality (i.e. satisfaction) of social support can influence the impact (103). The ‘social support (unspecified)’ BCT extracted from our analysis was mainly referring to the use of cognitive behavioral therapy or motivational interviewing techniques. Therefore, social support coming from the intervention facilitator. Fidelity assessment can indicate the amount and quality of psychological techniques delivered (alluding to the quality of social support from facilitators also), however, the minority of studies in the review reported fidelity assessment.

Problem solving techniques were developed by D’Zurilla and Goldfried and work by alleviating psychological distress in response to a stressful event through improving coping skills (104). Problem solving is a learned behavior that involves generating strategies to overcome barriers to diabetes self-management, applying these strategies, then evaluating these strategies (105). Problem solving for people with type 2 diabetes has been found to improve self-efficacy, coping styles, and well-being (106); decrease in depressive symptoms (107); and improve HbA1c (107, 108). It is a useful technique for people with depressive symptoms and type 2 diabetes who have impaired problem-solving skills (109).

Goal setting aims to increase self-efficacy in self-managing type 2 diabetes (110). Goal setting theory suggests that if someone achieves their goal, then they experience success, but if they do not achieve their goal this leads to discontent (111). Goals should be specific in order to promote attainment (112), for example, setting “SMART” goals that are specific, measurable, attainable, realistic, and timely. Goal setting for people with type 2 diabetes is associated with improved self-management behaviors (110, 113), diabetes distress, depressive symptoms (114) and HbA1c (112).

### Strengths and Limitations

A strength of this study is, by identifying which smaller components of psychological interventions (BCTs) improve glycemic levels, this ensures future development of psychological interventions for people with type 2 diabetes is more likely to be successful in improving glycemic levels. Another strength is at least two researchers were involved in the research process with high levels of inter-reliability indicating consistency in screening full-text papers and coding BCTs. Therefore, there is confidence that other researchers could replicate these methods and obtain similar results. However, during data extraction phase of our study, some psychological intervention descriptions were unclear. It is possible that not all relevant BCTs were extracted due to lack of reporting, quality of studies in discussed in more detail elsewhere (5). There were many individual BCTs that were not coded or common across psychological interventions, interventionist should consider using the BCT taxonomy for guidance when designing novel interventions, to improve reporting of such interventions and to help examine which active ingredients lead to improving outcomes. Interventionists should also be aware that psychological interventions are more than a sum of its parts i.e. BCTs, therapeutic alliance cannot be measured using BCTs, therefore, the way in which BCTs are delivered in psychological interventions needs to be considered.

This analysis did not code which BCTs underpinned the control groups in the RCTs. Most studies reported usual care as the control condition with limited description of what this entailed; therefore, BCT coding would not have been possible in most cases. Lack of description in control conditions has been previously discussed in health psychological research, and steps need to be taken to understand the active ingredients of an interventions as well as control conditions (115).

In our analysis, we did not distinguish between the types of self-management target behavioral domains e.g. physical activity, diet, self-monitoring blood glucose, optimal medication-taking behavior etc. This is a potential limitation as not necessarily all behaviors have an equal effect on glycaemia (116). However, this would be difficult to disentangle, as it is common for studies of people with type 2 diabetes to target more than one self-management behavior. This study focused on glycemic levels as an outcome, analysis of psychological (depression, diabetes distress) or self-management (dietary, optimal medication-taking behavior etc.) outcomes may have yielded different results. Glycemic levels (HbA1c) was an inclusion criterion of the original review, Winkley et al. (5), self-management and psychological outcomes were not an inclusion criterion. Therefore, we were unable to conduct a secondary analysis with these other outcomes, as they would have not pooled together all relevant literature.

RCTs did not test individual BCTs in isolation and pooling these studies for meta-analysis does not control for confounders. Therefore, it is uncertain which specific active ingredients lead to improvements. Improved reporting of active ingredients and development of more sophisticated meta-analytic methods may help identify which intervention components are truly associated with specific outcomes (96). Examining the use of a set of broader combination of BCTs could guide future intervention development to maximize intervention effects.

## Conclusions

This analysis was the first to determine which BCTs underpin psychological interventions targeting glycemic levels for people with type 2 diabetes. Future research to develop psychological interventions for people with type 2 diabetes should define BCTs in the psychological intervention design process, conduct fidelity assessment of interventionists, and ensure consistent reporting of BCTs. These steps would help to identify the specific active ingredients of a successful psychological interventions to improve glycemic levels for people with type 2 diabetes.

## Data Availability Statement

The raw data supporting the conclusions of this article will be made available by the authors, without undue reservation.

## Author Contributions

RU, KI, and KW contributed to the conception and design of the study. RU and DO performed BCT coding and data extraction. RU performed the statistical analysis and wrote the first draft of the manuscript. All authors contributed to the article and approved the submitted version.

## Funding

This paper presents independent research funded by the UK’s National for Health Research (NIHR) Health Technology Assessment (HTA) Evidence Synthesis Programme (reference: 12/213/10). The views expressed are those of the authors and not necessarily those of the NHS, the NIHR or the Department of Health and Social Care.

## Conflict of Interest

KW has served as a consultant or speaker for MSD and Valotech. KI has received honorarium for educational lectures for Jannssen, Sanofi, Eli Lilly and Novo Nordisk.

The remaining authors declare that the research was conducted in the absence of any commercial or financial relationships that could be construed as a potential conflict of interest.
