# Supplementary material for: The Effectiveness of Behavior Change Techniques Underpinning Psychological Interventions to Improve Glycemic Levels for Adults With Type 2 Diabetes: A Meta-Analysis
Source: Front Clin Diabetes Healthc. 2021 Jul 12;2:699038. doi: 10.3389/fcdhc.2021.699038 (PMC10012110; doi:10.3389/fcdhc.2021.699038)
Supplement: Supplementary file 1 [file Table_1.docx]

| Table S1- *Example of BCT data extraction* | |
| --- | --- |
| **BCT label** | **Example of BCT extracted from intervention descriptions** |
| **1.1 Goal setting (behaviour)** | *“the health coaches will work with the participant to establish a goal for behaviour change”*  *“encouraged to find their own goals and make them concrete and attainable”* |
| **1.2 Problem solving** | *“discussion of specific problems and developing techniques for dealing with them”*  *“Identify personal barriers and facilitators to lifestyle change”* |
| **1.3 Goal setting (outcome)** | *“encouraged to achieve moderate weight loss of 5–10% of initial body weight; A target of at least 210 min/week (30min every day) of moderate intensity planned aerobic activity was recommended”*  *"encouraged to discuss diabetes-specific goals, such as HbA1c target values"* |
| **1.4 Action planning** | *"develop written personalized action plans to improve self-management"*  *“...patients were instructed to wear the pedometer throughout waking hours for seven consecutive days.”* |
| **1.5 Review of behaviour goal(s)** | *"The goals formulated during the first visit are evaluated”*  *“goals formulated during the second visit are evaluated and new goals are formulated"* |
| **2.2 Feedback on behaviour** | *“the patient's change in behavior over the previous three months was discussed”*  *“given feedback and suggestions for improvement to ensure desired effectiveness criteria were met; Personalized feedback on self-care activities”* |
| **2.3 Self-monitoring of behaviour** | *“Monitoring of blood glucose levels and tracking dietary and physical activity behaviors”*  *“self-monitoring with regard to diabetes-relevant behavioral strategies (e.g., nutrition, physical activity, foot care”* |
| **2.4 Self-monitoring of outcome(s) of behaviour** | *“sent a pedometer, and were instructed to wear it and record total daily step-counts in a log for 3 consecutive days within the week”*  *“Participants were provided with a pedometer and a set of digital scales.”* |
| **3.1 Social support (unspecified)** | *“On the forum patients were encouraged to exchange views and ideas on diabetes self-care and discuss their progress in the management.”*  *“cognitive behavioural group intervention program”*  *“motivational interviewing”* |
| **3.3 Social support (emotional)** | *“allowed to bring along one significant other person as a co-participant”*  *“family members were encouraged to help with the monitoring of medication adherence”* |
| **4.1 Instruction on how to perform the behaviour** | *“Participants were also provided with a glucose meter with ten strips and were instructed in their use.”*  *“detailed instructions about how to accurately take measurements and record them in a DM diary.”* |
| **6.1 Demonstration of the behaviour** | *“guided again through the body scan”*  *“Modelling”* |
| **8.1 Behavioural practice/rehearsal** | *“a relaxation technique is taught to and practiced by the participants.”*  *“asked to practice the bodyscan on five occasions in the following week.”* |
| **8.7 Graded tasks** | *“Gradual increases in physical activity were promoted with an ultimate goal of at least 150 min/week”*  *“instructed to progress gradually from 20minutes, 4days/ week to 60minutes, 5 to 6days/week.”* |
| **9.2 Pros and cons** | *“exploring costs and benefits of changing the behaviors”*  *“Discussion of benefits of and barriers to increase PA and decrease sedentary behaviour—decisional balance”* |
| **10.3 Non-specific incentive** | *“the entire process was reviewed, emphasizing participant achievements”*  *“The researcher provided support and reinforcement to the participants to maintain their self-management.”* |
| **11.2 Reduce negative emotions** | *“encouraged to maintain their practice of stress management and relaxation skills.”*  *“learned about the link between diabetes and mood and ways to influence impaired mood with cognitive techniques”* |
| **12.5 Adding objects to the environment** | *“provided with a glucose monitor, strips, and lancet(s)”*  *“...all patients were sent an Omron HJ-720 ITC pedometer with a built-in clock and electronic memory.”* |
| **13.2 Framing/reframing** | *“Cognitive restructuring”* |
